# Crystal structure of (9*S*,10*S*)-10-eth­oxy-9-hy­droxy-6,6,9-trimethyl-3-pentyl-7,8,9,10-tetra­hydro-6*H*-benzo[*c*]chromen-1-yl 4-methyl­benzene­sulfonate

**DOI:** 10.1107/S2056989015024044

**Published:** 2015-12-24

**Authors:** Waseem Gul, Ahmed Galal, Mahmoud A. ElSohly, Paulo Carvalho

**Affiliations:** a National Center for Natural Products Research, The University of Mississippi, University, MS 38677, USA; b El Sohly Laboratories Inc., 5 Industrial Park Drive, Oxford, MS 38655, USA; c National Center for Natural Products Research, Department of Pharmaceutics, School of Pharmacy, The University of Mississippi, University, MS 38677, USA; d School of Pharmacy, Notre Dame of Maryland University, 4701 North Charles Street, 21210, Baltimore, MD, USA

**Keywords:** crystal structure, hydrogen bonding, Δ^9^–THC tosyl­ate, photooxygenation

## Abstract

In the structure of the title compound, C_30_H_40_O_6_S, the cyclo­hexene and heterocyclic rings are linked by a double bond. The cyclo­hexene ring has a half-chair conformation (the methyl­ene group adjacent to the hy­droxy substituent lies above the remaining atoms) and the hy­droxy and eth­oxy groups have equatorial and bis­ectional dispositions, respectively. The heterocyclic ring has an envelope conformation (with the CMe_2_ C atom being the flap). The dihedral angle between the aromatic rings is 53.88 (10)°. A long intra­molecular C—H⋯S inter­action is noted. In the mol­ecular packing, hy­droxy-O—H⋯O(sulfonate) hydrogen bonds lead to a helical chain along [010]. Connections between chains are of the type methyl-C—H⋯O(sulfonate) and lead to supra­molecular layers that lie parallel to (001). The studied crystal was an inversion twin.

## Related literature   

For Δ^9^–THC tosyl­ate, see: Ducker (2004 ▸); Gul *et al.* (2008 ▸). For a related process of photooxygenation, see: Motoyoshiya *et al.* (1999 ▸); Griesbeck *et al.* (2014 ▸). For unusually long sulfur hydrogen bonding, see: Huang *et al.* (2009 ▸).
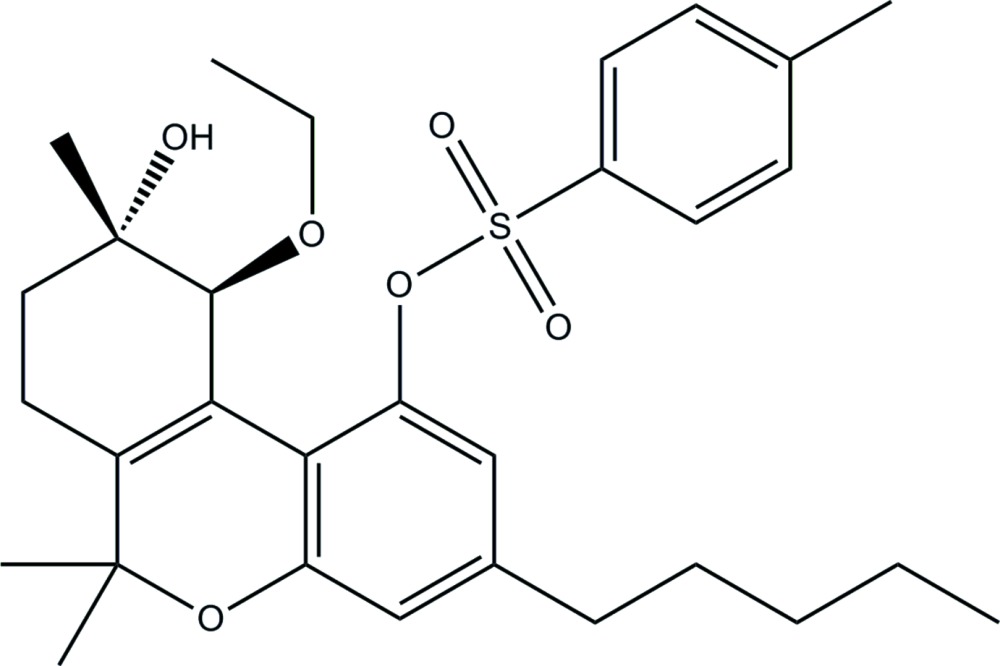


## Experimental   

### Crystal data  

C_30_H_40_O_6_S
*M* *_r_* = 528.68
Monoclinic, 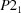

*a* = 9.909 (1) Å
*b* = 10.2373 (10) Å
*c* = 13.8402 (10) Å
β = 101.00 (1)°
*V* = 1378.2 (2) Å^3^
*Z* = 2
Cu *K*α radiation
μ = 1.38 mm^−1^
*T* = 173 K
0.23 × 0.20 × 0.19 mm

### Data collection  

Bruker SMART CCD area-detector diffractometer
21062 measured reflections
4984 independent reflections
4834 reflections with *I* > 2σ(*I*)
*R*_int_ = 0.025

### Refinement  

*R*[*F* ^2^ > 2σ(*F* ^2^)] = 0.025
*wR*(*F* ^2^) = 0.066
*S* = 1.02
4984 reflections
342 parameters
1 restraint
H-atom parameters constrained
Δρ_max_ = 0.26 e Å^−3^
Δρ_min_ = −0.28 e Å^−3^

### 

Data collection: *APEX2* (Bruker, 2014 ▸); cell refinement: *SAINT* (Bruker, 2000 ▸); data reduction: *SAINT*; program(s) used to solve structure: *SHELXS97* (Sheldrick, 2008 ▸); program(s) used to refine structure: *SHELXL2014*/7 (Sheldrick, 2015 ▸); molecular graphics: *Mercury* (Macrae *et al.*, 2008 ▸), *ORTEP-3 for Windows* (Farrugia, 2012 ▸), *POV-RAY* (Cason, 2003 ▸); software used to prepare material for publication: *enCIFer* (Allen *et al.*, 2004 ▸), *publCIF* (Westrip, 2010 ▸).

## Supplementary Material

Crystal structure: contains datablock(s) I, New_Global_Publ_Block. DOI: 10.1107/S2056989015024044/tk5416sup1.cif

Structure factors: contains datablock(s) I. DOI: 10.1107/S2056989015024044/tk5416Isup2.hkl

**Fig. 1.**
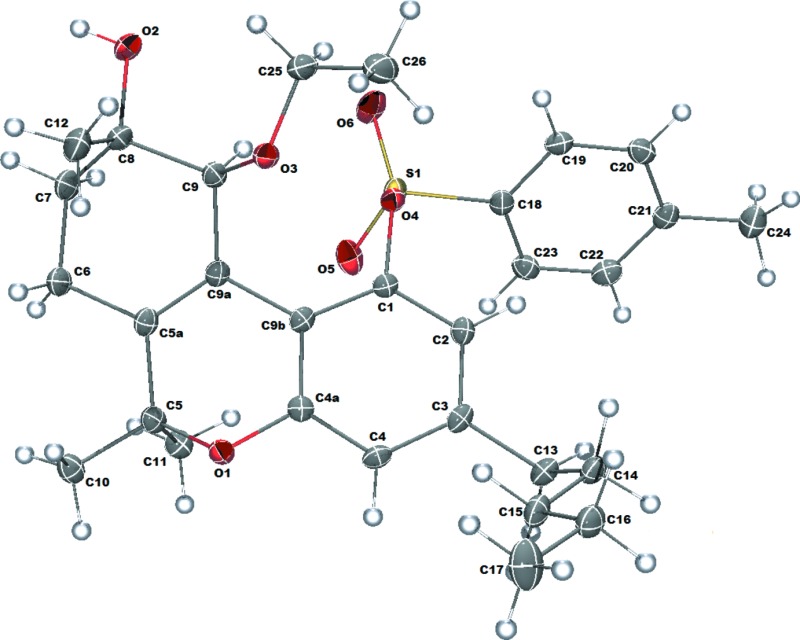
Plot of the molecular structure of the title compound with the atom-labelling scheme. Displacement ellipsoids are drawn at the 50% probability level.

. DOI: 10.1107/S2056989015024044/tk5416fig1.tif
Plot of the mol­ecular structure of the title compound with the atom-labelling scheme. Displacement ellipsoids are drawn at the 50% probability level.

**Fig. 2.**
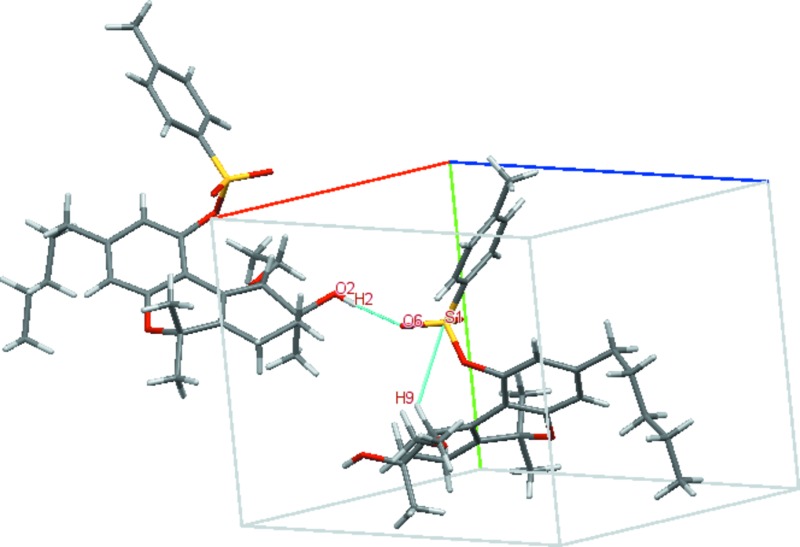
Partial plot of the unit-cell contents of the title compound, showing an O6—H2···O2 intermolecular H atoms bond and one long, intramolecular C9—H9···S1 hydrogen bond, both represented by light blue lines.

. DOI: 10.1107/S2056989015024044/tk5416fig2.tif
Partial plot of the unit cell contents of the title compound, showing an O6—H2⋯O2 inter­molecular hydrogens bond and one long, intra­molecular C9—H9⋯S1 hydrogen bond, both represented by light blue lines.

CCDC reference: 1442416

Additional supporting information:  crystallographic information; 3D view; checkCIF report

## Figures and Tables

**Table 1:** Hydrogen-bond geometry (Å, °)

*D*—H⋯*A*	*D*—H	H⋯*A*	*D*⋯*A*	*D*—H⋯*A*
C9—H9⋯S1	0.98	2.94	3.687 (2)	134
C10—H10*B*⋯O5^i^	0.96	2.57	3.459 (2)	154
O2—H2⋯O6^ii^	0.82	2.22	3.014 (2)	165

Symmetry codes: (i) 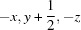; (ii) 
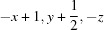
.

## supplementary crystallographic information

### S1. Experimental

Δ^9^–THC tosyl­ate was submitted to photooxygenation in di­chloro­methane/ethanol for 11 h and 30 min using *meso*-tetra­phenyl­porphine in the presence of oxygen and light generated by a regular, incandescent light bulb, yielding the title compound, which was crystallized from ethyl acetate: hexanes 1:9, producing needle-like crystals

### S1.1. Refinement

All H atoms were located in difference maps but were included in the model in the riding model approximation with O—H = 0.82 Å and C—H = 0.93–0.98 Å, and with *U*iso = 1.5*U*eq (O) for OH and *U*iso=1.2–1.5*U*eq(C).

### Crystal data

**Table:**

C_30_H_40_O_6_S	*F*(000) = 568
*M**_r_* = 528.68	*D*_x_ = 1.274 Mg m^−^^3^
Monoclinic, *P*2_1_	Cu *K*α radiation, λ = 1.54178 Å
*a* = 9.909 (1) Å	Cell parameters from 9903 reflections
*b* = 10.2373 (10) Å	θ = 4.6–68.1°
*c* = 13.8402 (10) Å	µ = 1.38 mm^−^^1^
β = 101.00 (1)°	*T* = 173 K
*V* = 1378.2 (2) Å^3^	Needle, colourless
*Z* = 2	0.23 × 0.20 × 0.19 mm

### Data collection

**Table:**

Bruker SMART CCD area-detector diffractometer	*R*_int_ = 0.025
Radiation source: X-ray	θ_max_ = 68.2°, θ_min_ = 3.3°
φ and ω scans	*h* = −11→11
21062 measured reflections	*k* = −12→12
4984 independent reflections	*l* = −16→16
4834 reflections with *I* > 2σ(*I*)	

### Refinement

**Table:**

Refinement on *F*^2^	Hydrogen site location: inferred from neighbouring sites
Least-squares matrix: full	H-atom parameters constrained
*R*[*F*^2^ > 2σ(*F*^2^)] = 0.025	*w* = 1/[σ^2^(*F*_o_^2^) + (0.0429*P*)^2^ + 0.1558*P*] where *P* = (*F*_o_^2^ + 2*F*_c_^2^)/3
*wR*(*F*^2^) = 0.066	(Δ/σ)_max_ < 0.001
*S* = 1.02	Δρ_max_ = 0.26 e Å^−^^3^
4984 reflections	Δρ_min_ = −0.28 e Å^−^^3^
342 parameters	Absolute structure: Twinning involves inversion, so Flack parameter cannot be determined
1 restraint	

### Special details

**Table:**

Geometry. All e.s.d.'s (except the e.s.d. in the dihedral angle between two l.s. planes) are estimated using the full covariance matrix. The cell e.s.d.'s are taken into account individually in the estimation of e.s.d.'s in distances, angles and torsion angles; correlations between e.s.d.'s in cell parameters are only used when they are defined by crystal symmetry. An approximate (isotropic) treatment of cell e.s.d.'s is used for estimating e.s.d.'s involving l.s. planes.
Refinement. Refined as a 2-component inversion twin.

### Fractional atomic coordinates and isotropic or equivalent isotropic displacement parameters (Å^2^)

**Table:**

	*x*	*y*	*z*	*U*_iso_*/*U*_eq_	
S1	0.33896 (5)	0.44735 (5)	0.19766 (3)	0.01720 (12)	
O1	0.01053 (14)	0.87882 (14)	0.24328 (10)	0.0163 (3)	
O2	0.54967 (16)	0.84850 (17)	0.05710 (11)	0.0269 (4)	
H2	0.5600	0.8838	0.0058	0.040*	
O3	0.47179 (14)	0.82165 (14)	0.25792 (10)	0.0174 (3)	
O4	0.37762 (14)	0.56548 (14)	0.27376 (10)	0.0158 (3)	
O5	0.20422 (16)	0.46627 (15)	0.14151 (10)	0.0247 (3)	
O6	0.45457 (17)	0.44066 (17)	0.15025 (11)	0.0269 (3)	
C1	0.2680 (2)	0.6273 (2)	0.30887 (14)	0.0144 (4)	
C2	0.2306 (2)	0.5743 (2)	0.39227 (14)	0.0156 (4)	
H2A	0.2785	0.5035	0.4240	0.019*	
C3	0.1205 (2)	0.6281 (2)	0.42811 (14)	0.0163 (4)	
C4	0.0496 (2)	0.7316 (2)	0.37639 (14)	0.0164 (4)	
H4	−0.0246	0.7688	0.3986	0.020*	
C4A	0.0882 (2)	0.78021 (19)	0.29185 (14)	0.0147 (4)	
C5	0.0027 (2)	0.8815 (2)	0.13601 (14)	0.0169 (4)	
C5A	0.1471 (2)	0.8735 (2)	0.11459 (14)	0.0170 (4)	
C6	0.1735 (2)	0.9385 (3)	0.02247 (14)	0.0228 (4)	
H6A	0.1767	1.0324	0.0319	0.027*	
H6B	0.0984	0.9190	−0.0316	0.027*	
C7	0.3085 (2)	0.8924 (3)	−0.00372 (15)	0.0255 (5)	
H7A	0.2977	0.8036	−0.0284	0.031*	
H7B	0.3309	0.9474	−0.0555	0.031*	
C8	0.4260 (2)	0.8973 (2)	0.08512 (15)	0.0203 (4)	
C9	0.3933 (2)	0.7990 (2)	0.16179 (14)	0.0164 (4)	
H9	0.4138	0.7108	0.1410	0.020*	
C9A	0.2436 (2)	0.80314 (19)	0.17329 (14)	0.0150 (4)	
C9B	0.2036 (2)	0.7346 (2)	0.25771 (14)	0.0148 (4)	
C10	−0.0683 (2)	1.0112 (2)	0.10561 (15)	0.0201 (4)	
H10A	−0.1549	1.0139	0.1269	0.030*	
H10B	−0.0834	1.0196	0.0353	0.030*	
H10C	−0.0112	1.0816	0.1354	0.030*	
C11	−0.0845 (2)	0.7666 (2)	0.08950 (15)	0.0205 (4)	
H11A	−0.0413	0.6862	0.1141	0.031*	
H11B	−0.0926	0.7697	0.0193	0.031*	
H11C	−0.1742	0.7718	0.1058	0.031*	
C12	0.4513 (3)	1.0345 (2)	0.12656 (18)	0.0277 (5)	
H12A	0.5337	1.0353	0.1760	0.042*	
H12B	0.3749	1.0612	0.1553	0.042*	
H12C	0.4612	1.0937	0.0746	0.042*	
C13	0.0733 (2)	0.5677 (2)	0.51606 (15)	0.0195 (4)	
H13A	0.0891	0.4743	0.5152	0.023*	
H13B	−0.0250	0.5813	0.5089	0.023*	
C14	0.1441 (2)	0.6214 (2)	0.61665 (15)	0.0197 (4)	
H14A	0.1119	0.5729	0.6680	0.024*	
H14B	0.2424	0.6073	0.6246	0.024*	
C15	0.1177 (2)	0.7662 (2)	0.62955 (15)	0.0215 (4)	
H15A	0.0193	0.7816	0.6137	0.026*	
H15B	0.1594	0.8151	0.5829	0.026*	
C16	0.1730 (2)	0.8183 (2)	0.73286 (15)	0.0223 (5)	
H16A	0.2713	0.8030	0.7495	0.027*	
H16B	0.1302	0.7714	0.7799	0.027*	
C17	0.1449 (3)	0.9644 (3)	0.74043 (16)	0.0333 (6)	
H17A	0.1857	1.0110	0.6931	0.050*	
H17B	0.1838	0.9946	0.8055	0.050*	
H17C	0.0474	0.9793	0.7275	0.050*	
C18	0.3399 (2)	0.31006 (19)	0.27416 (15)	0.0159 (4)	
C19	0.4624 (2)	0.2741 (2)	0.33485 (15)	0.0185 (4)	
H19	0.5419	0.3228	0.3360	0.022*	
C20	0.4641 (2)	0.1648 (2)	0.39354 (15)	0.0202 (4)	
H20	0.5454	0.1408	0.4353	0.024*	
C21	0.3458 (2)	0.0896 (2)	0.39119 (15)	0.0186 (4)	
C22	0.2245 (2)	0.1286 (2)	0.32967 (16)	0.0214 (5)	
H22	0.1449	0.0797	0.3275	0.026*	
C23	0.2205 (2)	0.2395 (2)	0.27151 (15)	0.0193 (4)	
H23	0.1388	0.2659	0.2314	0.023*	
C24	0.3507 (2)	−0.0315 (2)	0.45314 (16)	0.0265 (5)	
H24A	0.3732	−0.1053	0.4165	0.040*	
H24B	0.2625	−0.0452	0.4707	0.040*	
H24C	0.4193	−0.0213	0.5118	0.040*	
C25	0.6098 (2)	0.7735 (2)	0.27438 (16)	0.0229 (5)	
H25A	0.6684	0.8324	0.2461	0.027*	
H25B	0.6129	0.6880	0.2447	0.027*	
C26	0.6567 (2)	0.7654 (3)	0.38428 (17)	0.0305 (5)	
H26A	0.6577	0.8513	0.4122	0.046*	
H26B	0.7476	0.7289	0.3991	0.046*	
H26C	0.5948	0.7107	0.4116	0.046*	

### Atomic displacement parameters (Å^2^)

**Table:**

	*U*^11^	*U*^22^	*U*^33^	*U*^12^	*U*^13^	*U*^23^
S1	0.0244 (3)	0.0168 (2)	0.0111 (2)	0.0027 (2)	0.00518 (17)	−0.00065 (18)
O1	0.0180 (7)	0.0179 (7)	0.0134 (6)	0.0035 (6)	0.0041 (5)	−0.0009 (5)
O2	0.0259 (8)	0.0394 (9)	0.0193 (7)	0.0076 (7)	0.0136 (6)	0.0107 (7)
O3	0.0158 (7)	0.0233 (7)	0.0133 (7)	0.0012 (6)	0.0030 (5)	0.0015 (5)
O4	0.0170 (7)	0.0160 (7)	0.0154 (7)	0.0019 (6)	0.0056 (6)	0.0006 (5)
O5	0.0327 (8)	0.0238 (8)	0.0150 (6)	0.0034 (7)	−0.0024 (6)	−0.0019 (6)
O6	0.0393 (9)	0.0238 (7)	0.0222 (7)	0.0036 (7)	0.0177 (7)	−0.0009 (7)
C1	0.0149 (10)	0.0162 (9)	0.0121 (9)	−0.0014 (8)	0.0023 (8)	−0.0030 (7)
C2	0.0178 (10)	0.0152 (9)	0.0128 (9)	−0.0019 (8)	0.0007 (8)	−0.0005 (7)
C3	0.0196 (10)	0.0183 (9)	0.0110 (9)	−0.0065 (8)	0.0032 (8)	−0.0029 (7)
C4	0.0162 (10)	0.0197 (10)	0.0148 (9)	−0.0015 (8)	0.0065 (8)	−0.0037 (8)
C4A	0.0150 (9)	0.0149 (9)	0.0134 (9)	−0.0020 (8)	0.0009 (7)	−0.0034 (7)
C5	0.0186 (10)	0.0206 (9)	0.0111 (9)	0.0027 (8)	0.0017 (8)	−0.0006 (8)
C5A	0.0212 (11)	0.0168 (10)	0.0134 (9)	−0.0007 (8)	0.0040 (8)	−0.0003 (8)
C6	0.0218 (10)	0.0306 (11)	0.0163 (9)	0.0048 (10)	0.0042 (8)	0.0081 (9)
C7	0.0275 (12)	0.0356 (12)	0.0147 (9)	0.0060 (10)	0.0075 (9)	0.0089 (9)
C8	0.0195 (11)	0.0252 (11)	0.0183 (10)	0.0024 (9)	0.0090 (8)	0.0051 (8)
C9	0.0188 (10)	0.0187 (10)	0.0124 (9)	0.0026 (8)	0.0045 (8)	0.0005 (7)
C9A	0.0197 (10)	0.0149 (9)	0.0111 (9)	0.0005 (8)	0.0047 (8)	−0.0024 (7)
C9B	0.0163 (10)	0.0170 (9)	0.0109 (9)	−0.0025 (8)	0.0022 (7)	−0.0025 (7)
C10	0.0226 (11)	0.0206 (10)	0.0164 (9)	0.0045 (9)	0.0019 (8)	−0.0005 (8)
C11	0.0213 (11)	0.0214 (11)	0.0180 (9)	0.0004 (9)	0.0017 (8)	−0.0022 (8)
C12	0.0308 (13)	0.0252 (12)	0.0296 (12)	0.0010 (10)	0.0121 (10)	0.0091 (10)
C13	0.0213 (11)	0.0215 (10)	0.0172 (10)	−0.0026 (9)	0.0076 (8)	0.0008 (8)
C14	0.0194 (11)	0.0264 (11)	0.0143 (9)	−0.0011 (9)	0.0057 (8)	0.0035 (8)
C15	0.0240 (11)	0.0263 (11)	0.0143 (9)	−0.0029 (9)	0.0041 (8)	0.0044 (8)
C16	0.0226 (11)	0.0291 (12)	0.0152 (10)	−0.0007 (9)	0.0038 (8)	0.0025 (8)
C17	0.0470 (14)	0.0297 (13)	0.0193 (10)	−0.0033 (12)	−0.0036 (10)	−0.0003 (10)
C18	0.0205 (10)	0.0143 (9)	0.0139 (9)	0.0028 (8)	0.0055 (8)	−0.0023 (7)
C19	0.0154 (10)	0.0229 (11)	0.0178 (9)	−0.0017 (8)	0.0046 (8)	−0.0023 (8)
C20	0.0186 (10)	0.0247 (11)	0.0169 (9)	0.0033 (9)	0.0024 (8)	−0.0015 (8)
C21	0.0236 (11)	0.0180 (10)	0.0159 (9)	0.0007 (8)	0.0077 (8)	−0.0032 (8)
C22	0.0211 (11)	0.0194 (10)	0.0247 (11)	−0.0049 (8)	0.0071 (9)	−0.0048 (8)
C23	0.0179 (10)	0.0219 (11)	0.0177 (9)	0.0029 (9)	0.0021 (8)	−0.0025 (8)
C24	0.0340 (12)	0.0235 (12)	0.0231 (10)	−0.0001 (10)	0.0080 (9)	0.0020 (9)
C25	0.0169 (10)	0.0309 (12)	0.0220 (11)	0.0009 (9)	0.0064 (9)	0.0040 (9)
C26	0.0192 (11)	0.0478 (15)	0.0237 (11)	0.0032 (11)	0.0020 (9)	0.0053 (11)

### Geometric parameters (Å, º)

**Table:**

S1—O5	1.4240 (16)	C11—H11B	0.9600
S1—O6	1.4264 (15)	C11—H11C	0.9600
S1—O4	1.6014 (15)	C12—H12A	0.9600
S1—C18	1.759 (2)	C12—H12B	0.9600
O1—C4A	1.366 (3)	C12—H12C	0.9600
O1—C5	1.472 (2)	C13—C14	1.536 (3)
O2—C8	1.443 (2)	C13—H13A	0.9700
O2—H2	0.8200	C13—H13B	0.9700
O3—C9	1.426 (2)	C14—C15	1.521 (3)
O3—C25	1.431 (3)	C14—H14A	0.9700
O4—C1	1.421 (2)	C14—H14B	0.9700
C1—C2	1.388 (3)	C15—C16	1.527 (3)
C1—C9B	1.394 (3)	C15—H15A	0.9700
C2—C3	1.395 (3)	C15—H15B	0.9700
C2—H2A	0.9300	C16—C17	1.528 (3)
C3—C4	1.392 (3)	C16—H16A	0.9700
C3—C13	1.517 (3)	C16—H16B	0.9700
C4—C4A	1.391 (3)	C17—H17A	0.9600
C4—H4	0.9300	C17—H17B	0.9600
C4A—C9B	1.398 (3)	C17—H17C	0.9600
C5—C5A	1.518 (3)	C18—C23	1.380 (3)
C5—C10	1.523 (3)	C18—C19	1.387 (3)
C5—C11	1.526 (3)	C19—C20	1.381 (3)
C5A—C9A	1.340 (3)	C19—H19	0.9300
C5A—C6	1.505 (3)	C20—C21	1.397 (3)
C6—C7	1.526 (3)	C20—H20	0.9300
C6—H6A	0.9700	C21—C22	1.393 (3)
C6—H6B	0.9700	C21—C24	1.503 (3)
C7—C8	1.524 (3)	C22—C23	1.388 (3)
C7—H7A	0.9700	C22—H22	0.9300
C7—H7B	0.9700	C23—H23	0.9300
C8—C12	1.520 (3)	C24—H24A	0.9600
C8—C9	1.541 (3)	C24—H24B	0.9600
C9—C9A	1.523 (3)	C24—H24C	0.9600
C9—H9	0.9800	C25—C26	1.506 (3)
C9A—C9B	1.480 (3)	C25—H25A	0.9700
C10—H10A	0.9600	C25—H25B	0.9700
C10—H10B	0.9600	C26—H26A	0.9600
C10—H10C	0.9600	C26—H26B	0.9600
C11—H11A	0.9600	C26—H26C	0.9600
			
O5—S1—O6	120.48 (9)	H11A—C11—H11C	109.5
O5—S1—O4	109.74 (8)	H11B—C11—H11C	109.5
O6—S1—O4	103.09 (9)	C8—C12—H12A	109.5
O5—S1—C18	109.51 (10)	C8—C12—H12B	109.5
O6—S1—C18	109.04 (10)	H12A—C12—H12B	109.5
O4—S1—C18	103.56 (8)	C8—C12—H12C	109.5
C4A—O1—C5	115.24 (15)	H12A—C12—H12C	109.5
C8—O2—H2	109.5	H12B—C12—H12C	109.5
C9—O3—C25	115.27 (15)	C3—C13—C14	115.09 (17)
C1—O4—S1	117.32 (12)	C3—C13—H13A	108.5
C2—C1—C9B	124.13 (19)	C14—C13—H13A	108.5
C2—C1—O4	116.98 (18)	C3—C13—H13B	108.5
C9B—C1—O4	118.88 (17)	C14—C13—H13B	108.5
C1—C2—C3	119.5 (2)	H13A—C13—H13B	107.5
C1—C2—H2A	120.2	C15—C14—C13	113.32 (18)
C3—C2—H2A	120.2	C15—C14—H14A	108.9
C4—C3—C2	118.04 (18)	C13—C14—H14A	108.9
C4—C3—C13	121.37 (19)	C15—C14—H14B	108.9
C2—C3—C13	120.41 (19)	C13—C14—H14B	108.9
C4A—C4—C3	120.78 (18)	H14A—C14—H14B	107.7
C4A—C4—H4	119.6	C14—C15—C16	114.31 (18)
C3—C4—H4	119.6	C14—C15—H15A	108.7
O1—C4A—C4	117.27 (18)	C16—C15—H15A	108.7
O1—C4A—C9B	120.06 (17)	C14—C15—H15B	108.7
C4—C4A—C9B	122.64 (19)	C16—C15—H15B	108.7
O1—C5—C5A	109.00 (16)	H15A—C15—H15B	107.6
O1—C5—C10	103.17 (15)	C15—C16—C17	111.65 (19)
C5A—C5—C10	113.29 (17)	C15—C16—H16A	109.3
O1—C5—C11	109.13 (16)	C17—C16—H16A	109.3
C5A—C5—C11	110.76 (17)	C15—C16—H16B	109.3
C10—C5—C11	111.16 (16)	C17—C16—H16B	109.3
C9A—C5A—C6	122.14 (18)	H16A—C16—H16B	108.0
C9A—C5A—C5	120.03 (17)	C16—C17—H17A	109.5
C6—C5A—C5	117.68 (17)	C16—C17—H17B	109.5
C5A—C6—C7	111.51 (18)	H17A—C17—H17B	109.5
C5A—C6—H6A	109.3	C16—C17—H17C	109.5
C7—C6—H6A	109.3	H17A—C17—H17C	109.5
C5A—C6—H6B	109.3	H17B—C17—H17C	109.5
C7—C6—H6B	109.3	C23—C18—C19	121.6 (2)
H6A—C6—H6B	108.0	C23—C18—S1	119.73 (16)
C8—C7—C6	111.56 (17)	C19—C18—S1	118.70 (16)
C8—C7—H7A	109.3	C20—C19—C18	118.86 (19)
C6—C7—H7A	109.3	C20—C19—H19	120.6
C8—C7—H7B	109.3	C18—C19—H19	120.6
C6—C7—H7B	109.3	C19—C20—C21	121.07 (19)
H7A—C7—H7B	108.0	C19—C20—H20	119.5
O2—C8—C12	109.50 (18)	C21—C20—H20	119.5
O2—C8—C7	109.20 (17)	C22—C21—C20	118.61 (19)
C12—C8—C7	112.3 (2)	C22—C21—C24	121.0 (2)
O2—C8—C9	105.08 (16)	C20—C21—C24	120.4 (2)
C12—C8—C9	112.64 (17)	C23—C22—C21	121.0 (2)
C7—C8—C9	107.81 (18)	C23—C22—H22	119.5
O3—C9—C9A	105.46 (15)	C21—C22—H22	119.5
O3—C9—C8	112.79 (17)	C18—C23—C22	118.9 (2)
C9A—C9—C8	113.00 (17)	C18—C23—H23	120.5
O3—C9—H9	108.5	C22—C23—H23	120.5
C9A—C9—H9	108.5	C21—C24—H24A	109.5
C8—C9—H9	108.5	C21—C24—H24B	109.5
C5A—C9A—C9B	117.76 (18)	H24A—C24—H24B	109.5
C5A—C9A—C9	123.25 (18)	C21—C24—H24C	109.5
C9B—C9A—C9	118.82 (17)	H24A—C24—H24C	109.5
C1—C9B—C4A	114.59 (18)	H24B—C24—H24C	109.5
C1—C9B—C9A	127.53 (18)	O3—C25—C26	106.32 (17)
C4A—C9B—C9A	117.88 (18)	O3—C25—H25A	110.5
C5—C10—H10A	109.5	C26—C25—H25A	110.5
C5—C10—H10B	109.5	O3—C25—H25B	110.5
H10A—C10—H10B	109.5	C26—C25—H25B	110.5
C5—C10—H10C	109.5	H25A—C25—H25B	108.7
H10A—C10—H10C	109.5	C25—C26—H26A	109.5
H10B—C10—H10C	109.5	C25—C26—H26B	109.5
C5—C11—H11A	109.5	H26A—C26—H26B	109.5
C5—C11—H11B	109.5	C25—C26—H26C	109.5
H11A—C11—H11B	109.5	H26A—C26—H26C	109.5
C5—C11—H11C	109.5	H26B—C26—H26C	109.5

### Hydrogen-bond geometry (Å, º)

**Table:**

*D*—H···*A*	*D*—H	H···*A*	*D*···*A*	*D*—H···*A*
C9—H9···S1	0.98	2.94	3.687 (2)	134
C10—H10*B*···O5^i^	0.96	2.57	3.459 (2)	154
O2—H2···O6^ii^	0.82	2.22	3.014 (2)	165

Symmetry codes: (i) −*x*, *y*+1/2, −*z*; (ii) −*x*+1, *y*+1/2, −*z*.
