# Supplementary figures and images for: Crystal structure of (9S,10S)-10-eth­oxy-9-hy­droxy-6,6,9-trimethyl-3-pentyl-7,8,9,10-tetra­hydro-6H-benzo[c]chromen-1-yl 4-methyl­benzene­sulfonate
Source: Acta Crystallogr E Crystallogr Commun. 2015 Dec 24;71(Pt 12):o1082–3. doi: 10.1107/S2056989015024044 (PMC4719990; doi:10.1107/S2056989015024044)

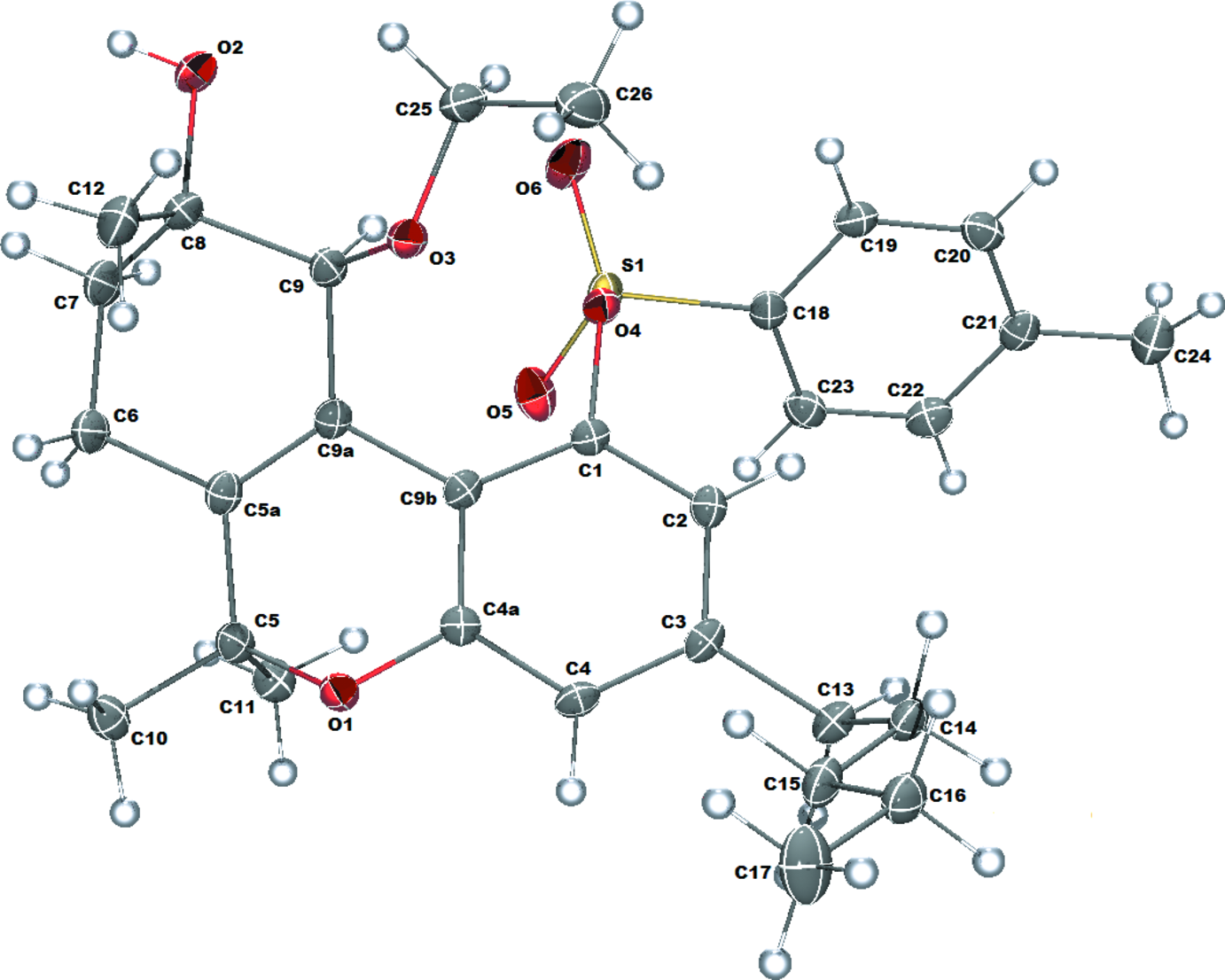

Supplement: Supplementary file 3 [file e-71-o1082-fig1.tif]

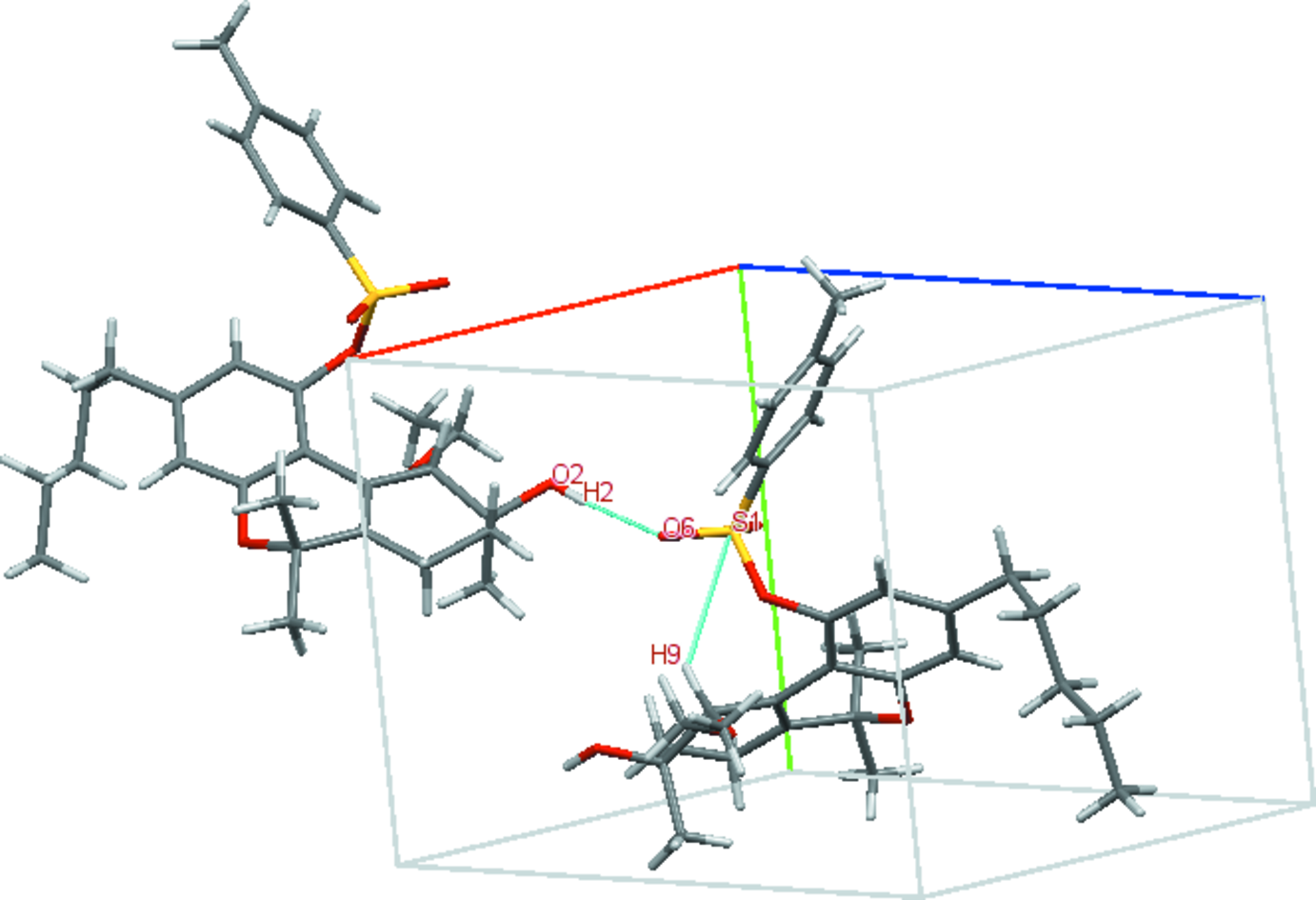

Supplement: Supplementary file 4 [file e-71-o1082-fig2.tif]
